# Effects of Cannabis Exposure on Adolescent Health and Development: A Narrative Review

**DOI:** 10.2174/0125899775273727231224185028

**Published:** 2024-01-15

**Authors:** Ruixuan Li, Feng Tao

**Affiliations:** 1 The Hockaday School, Dallas, Texas, USA;; 2 Department of Pediatrics, University of Texas Southwestern Medical Center, Dallas, Texas, USA;; 3 Department of Biomedical Sciences, Texas A&M University School of Dentistry, Dallas, Texas, USA

**Keywords:** Adolescence, brain, cannabis, cannabinoid receptor, substance use, legalization

## Abstract

Adolescence is an immature but adventurous time period of exploration. Due to rapid developments of the brain with unbalanced release of neurotransmitters, adolescents are prone to impulsivity that may carry out potentially dangerous behaviors. According to the Centers for Disease Control and Prevention, more than one-third of high school students have reported the use of cannabis or marijuana in 2019, and the trend has not declined since. Previous studies have shown that cannabis not only affects cognitive and social behaviors, but also produces psychological responses to stress. In this review, we have summarized recent studies on cannabis-produced effects during the unique period of adolescent development, and we have also briefly discussed the legalization of cannabis. Though there are slight differences between cannabis and marijuana, the major active component of them is tetrahydrocannabinol. We have used the term “cannabis” in this review. Cannabis use in adolescents causes structural and functional changes in the brain, increasing the probability of depression, which is also associated with other illicit substance use, and impairs education achievement. Given that cannabis use can cause detrimental harm to adolescents, it is suggested that adolescents should generally avoid using cannabis in a recreational manner. More preclinical and clinical studies are needed to provide detailed information for evidence-based decisions.

## 1 INTRODUCTION

Cannabis or marijuana is arguably one of the most controversial substances in the long history of all substance use. According to the National Institutes of Health, cannabis and marijuana describe different objects though often used interchangeably. The term “cannabis” refers to all products derived from the cannabis plant, while the term “marijuana” refers to specific parts of the same plant that contain significant amounts of the active ingredient tetrahydrocannabinol. The cannabis plant has a wide range of uses ranging from its roots to stems to leaves. For example, the roots can be used in medicine, the stem is known as hemp and can be used in textiles, and the leaves and flowers of the plant are more commonly known as marijuana [1]. To discuss all products coming from the cannabis plant, we have used the term “cannabis” in the following descriptions. Cannabis plants include male and female counterparts; the cannabis that is used or smoked comes from female plants that are capable of flowering, which usually also consist of a slender main stem and larger leaves [2]. While cannabis contains a variety of compounds, its psychoactive pharmacological component is tetrahydrocannabinol (THC). With a tri-cyclic 21-carbon structure, THC is a highly fat-soluble and volatile substance. THC may interact directly or indirectly with molecules and receptors in the brain, acting on a variety of signaling pathways and influencing neuronal transmission [3].

Different countries adopt different policies on cannabis use, which results in various perceptions and attitudes towards the substance among adolescents worldwide. Presently, cannabis use is legalized in Canada, and an increasing number of states in the United States have legalized the medical use of cannabis, its recreational use, or both. Coupled with the particularity of adolescent development, cannabis use among adolescents has been increasing, because teenagers in the period of physical and mental development become more prone to impulsive risk-taking behaviors. According to the Centers for Disease Control and Prevention, about one-third of teens have used cannabis at least once in their life. Consequently, cannabis use causes serious effects on psychological behavioral development in adolescents [4, 5]. In this review, we have summarized recent studies on how cannabis use affects adolescents in terms of their health and development, and we have also briefly discussed the legalization of cannabis.

## 2 METHODS

We searched Pubmed, Google Scholar, and EMBASE databases using the following keywords: “cannabis”, “adolescence”, “cannabinoid receptor”, and “substance use”. More than half of the references have been published within ten years. We carefully read the relevant references and summarized cannabis-produced effects during the unique period of adolescent development in those studies.

## 3 PHARMACOLOGY OF CANNABIS

Cannabis contains active and inactive ingredients; its active ingredients include psychoactive and non-psychoactive components. The former consists of delta-8 and delta-9 THC, while the latter consists of cannabidiol (CBD). The activity of delta-8 THC is weaker than that of delta-9 THC [3]. THC acts through endocannabinoid receptors to exert its pharmacological effects [2, 6, 7], and these receptors are G protein-coupled receptors, including cannabinoid receptors 1 and 2 (CB1 and CB2). CB1 receptors are mainly found in the brain and are often referred to as the central receptor, though small CB1 receptors are also found in peripheral tissues. On the other hand, CB2 receptors are mainly found in the peripheral immune system, especially macrophages. CB2 is also expressed in the brain and involved in the microglial function [8]. Both CB1 and CB2 receptors can inhibit N-type calcium ion channels and adenylate cyclase activity when activated [6]. Since CB1 receptors are mainly expressed on the axon and terminals of neurons, especially the presynaptic membrane [8], they influence the release of neurotransmitters, such as gamma-aminobutyric acid, glutamate, and acetylcholine, when THC binds to CB1 receptors. However, when THC binds to CB2 receptors, it affects immune cells to secrete cytokines and inflammatory factors and then participates in regulating inflammatory responses [8, 9].

In addition to psychoactive THC, cannabis also has a non-psychoactive ingredient, CBD. CBD serves as an inverse agonist in regulating both CB1 and CB2 receptors non-competitively [10]. Moreover, some studies indicate that CBD regulates the function of CB receptors through allosteric binding sites [11-13]. Although it has been demonstrated that CBD has negative regulation or antagonistic effects on CB1 receptors, it remains unclear how CBD affects CB2 receptors. There are studies showing that CBD is an antagonist to CB2 receptors, while others indicate that it is a partial agonist [10, 14]. Previous studies have shown that when CB1 and CB2 receptors are activated, they can inhibit adenylyl cyclase, thereby reducing the production of cyclic adenosine monophosphate and further inhibiting the activity of protein kinase A [15, 16]. CB2 receptors mainly initiate mitogen-activated protein kinase kinase/extracellular signal-regulated kinase signaling after activation, while CB1 receptors mainly exert their effects through the phosphatidylinositol 3-kinase/protein kinase B signaling pathway [17, 18].

In fact, there is an endocannabinoid system in the body. Endogenous cannabinoids mainly include N-arachidonoyl ethanolamine (AEA, anandamide) and 2-arachidonoylglycerol (2-AG), which are derivatives of arachidonic acid and generally have lipid structures [19, 20]. The two endogenous cannabinoids have different affinities and agonistic effects on CB receptors. 2-AG activates CB1 and CB2 receptors, but with lower affinity, whereas AEA binds to and activates CB1 receptors, but not CB2 receptors [21, 22]. An additional difference between AEA and 2-AG is that AEA can activate the transient receptor potential vanilloid 1 (TRPV1) receptor, while 2-AG cannot activate the TRPV1 receptor [19, 20, 23]. A representation of different receptors through which exogenous and endogenous cannabinoids act is shown in Fig. (**1**).

## 4 ADVERSE OUTCOMES OF CANNABIS USE IN ADOLESCENTS

Cannabis use in adolescents often leads to mental and behavioral changes, which affect the quality of life in their adulthood. Previous studies reveal that cannabis use in this period is strongly associated with mood disorders, split personality, anxiety, behavioral and neurocognitive disorders, as well as increased susceptibility to dependence, resulting in dramatic impacts on emotional development and future adult life [24-26]. A 10-year prospective study of 1395 adolescents aged 14 to 17 has demonstrated that mood and anxiety disorders are tightly correlated with cannabis use [24].

### 4.1 Cannabis-caused Structural and Functional Changes in the Brain

Functional magnetic resonance imaging (fMRI) and diffusion tensor imaging (DTI) studies have shown that the network of neural connections between caudal anterior cingulate cortex and left dorsolateral prefrontal cortex as well as orbitofrontal cortex change over time in adolescents with cannabis use disorder, and young adults who have used cannabis before age 17 and continued to use heavily for 2 years until after reaching adulthood exhibit changes in many white matter regions and network connections in the brain [27, 28]. Analysis of DTI longitudinal fractional anisotropy and radical diffusion parameter measures showed cannabis users to have significant differences in the brain’s superior longitudinal tact, parietal lobe, superior frontal gyrus, and cingulate gyrus compared to controls, as well as poor performances in language learning [27, 28]. Furthermore, a relative decrease in CB1 receptor expression in cannabis-using adolescents has been found to be accompanied by reduced cortical thickness and altered brain function, especially in the frontoparietal network, as observed by positron emission tomography scanning and fMRI studies, although no behavioral abnormalities have been found to occur compared to normal controls [29, 30].

Cannabis use not only affects adolescent brain volume, but also exacerbates pre-existing vulnerabilities to schizophrenia-related brain changes in young adults suffering from schizophrenia, thereby aggravating the brain volume loss [31]. In a study on young males with recent-onset schizophrenia, cannabis use had no effect on cortical thickness, and it only altered the cortical surface area [32]. Interestingly, cannabis use in adults has different effects on brain structures than those in adolescents, and it is reported that bilateral hippocampus and amygdala volume reductions, but not cortex, occur in adults with cannabis use, and the reduction in the hippocampus is greater than that in the amygdala [33]. In contrast to other studies, however, a large-size youth study (9,498 youths, 8-22 years old) did not observe a strong relationship between changes in brain structure and occasional or frequent cannabis use among adolescents, and this study also showed that youth with frequent cannabis use may have less cortical thickness in the left prefrontal cortex [34]. Further studies are needed to determine whether adolescent cannabis use is directly associated with structural and functional changes in the brain.

### 4.2 Cannabis-caused Cognitive Dysfunction and Depression

Among the cannabis-induced neurological disorders in adolescents, cognitive dysfunction has received more attention [27, 28]. The onset of an ultra-high risk for psychosis is related to the age of cannabis exposure: the younger the age, the earlier the onset of psychosis [35]. An 18-month longitudinal study with resting-state fMRI imaging showed that young adults who used cannabis exhibited decreased functional connectivity between the anterior cingulate cortex and dorsolateral/orbitofrontal cortices over time, predicting cognitive functional deficits [28]. Animal studies have also confirmed cannabis exposure to impair cognitive behaviors and inhibitory/excitatory neuronal balance in the hippocampus [36, 37]. However, when further identifying frequent and occasional cannabis users among adolescents, researchers realized that it is not possible to generalize that cannabis use among youth would always lead to cognitive deficits, as one large-size study with 4,568 adolescents showed that although young frequent cannabis users do have mild executive and cognitive dysfunctions, occasional cannabis users display even better cognitive behaviors than normal non-users [38]. This finding is similar to that of another study with adult cannabis users, which speculated that THC may cause damage to brain function and neural networks in the hippocampus, but CBD could delay or attenuate the THC-produced brain damage [39]. Thus, CBD may have a neuroprotective effect on hippocampal damage and also play an important role in brain cognitive function and synaptic plasticity [40, 41].

Early cannabis exposure can increase the probability of depression. It has been reported that regular cannabis users are consistently more emotionally impaired than occasional cannabis users and non-users [42]. Adolescent cannabis exposure and depression may co-occur and thus enhance the onset of each other. According to a study on 1,606 teenagers in Canada, adolescents who used cannabis were 11 to 15 times more likely to continue using it over time while experiencing depression even with suicidal tendencies, where symptoms of depression and cannabis use were mutually enhancing [43]. Previous studies also showed that depression patients who abused cannabis had four times more symptoms in subsequent follow-up than depression patients without cannabis use and were prone to antisocial behavior and suicidal behavior [44-46]. Although adolescent cannabis use is strongly associated with the later development of depression, it does not mean that treating depression will reduce cannabis use in teenagers [44]. The reason for the strong relationship between cannabis use disorder and depression may be due to the fact that patients with depression control symptoms through smoking cannabis, or that there are common genetic or environmental factors for this comorbidity [47].

Additionally, cannabis use before adulthood increases antisocial behavior as well as traffic incidents in teenagers [48, 49]. This has been confirmed in a 3-year long-term follow-up study with 43,653 adults over the age of 18; it was found that cannabis use increased the risk and susceptibility to other substances, such as alcohol [25]. Regarding acute and chronic effects of cannabis use, accumulating evidence suggests that acute symptoms include anxiety, mental disorders, and even suicidal tendencies, while chronic symptoms include, but not limited to, mental illness, cognitive behavioral disorders, and other systemic diseases [50]. A summary of cannabis use-produced adverse outcomes is illustrated in Fig. (**2**).

### 4.3 Cannabis-induced Other Illicit Substance Use

Strong links between cannabis use and other illicit substance use have been considered as a “gateway” of cannabis to other drugs. Drugs with a gateway pattern are identified as a leading cause of subsequent drug use or increased likelihood of the onset of other drug use [51-54]. This can be seen in the following ways. Firstly, there is a clear chronological sequence between cannabis and other substance use, and the first-time use of other substances usually occurs after cannabis use [51, 52]. Secondly, the dose and frequency of cannabis exposure are closely related to the use of other illicit substances, such that regular cannabis users are 100 times more likely to be exposed to other drugs than non-users [51-53]. Thirdly, other substances, especially alcohol and tobacco, appear to have a gateway effect on developing cannabis use, which in turn acts as a gateway to other illicit substance use [52, 53]. Speaking of the relationship between cannabis and alcohol or nicotine use, an early survey on 1,325 10^th^ and 11^th^ graders in New York state followed them until they were around age 24 and revealed how alcohol or nicotine use increases the possibility of cannabis exposure, and also showed that cannabis use in turn can lead to an increased likelihood of using other drugs [54]. Further epidemiological surveys among adolescents and young adults 12 to 25 years old demonstrated that alcohol and tobacco first increase the use of cannabis, which then increases the likelihood of the use of cocaine, portraying the gateway impact of cannabis in adolescence from a human research perspective [55]. Moreover, the interaction between cannabis use and antidepressant medications also affects the physical and mental health of young people, as shown by a study on adolescents with depression who were more likely to develop cannabis use, and exposure to cannabis further affected teenagers and young adults who were taking psychotropic or mood-related medications [52, 54]. Although adults are not as susceptible to cannabis and other substance use as adolescents, a survey of a large number of adults identified a strong relationship between cannabis and other drug use, as high as 44.7% of cannabis users in a certain phase became users of other drugs [56]. In addition, cannabis use has been found to sensitize other dependence-prone possibilities, such as heroin [57]. This may be due to the fact that cannabis can act as a “gateway” that triggers and enhances the user's dependence and sensitivity to other drugs [48, 57].

Animal studies display that the “gateway” of cannabis impacts adolescence more than adulthood [58, 59]. In a rat study [58], young Sprague-Dawley rats were intraperitoneally injected with 1 mg/kg of THC per day at the stage of adolescence between postnatal 28 and 45 days. After adulthood (postnatal 90 days), the rats were given different dosages of cocaine, and their responses to the injection of cocaine were detected by locomotor activities. This study showed that exposure to THC enhanced the effect of cocaine on adolescent but not adult rats, suggesting that repeated use of cannabis in adolescence may increase susceptibility to other drugs in later adulthood [58]. In addition, when 6-week-old Lewis rats, prone to substance dependency, were exposed to THC twice daily for three days, the rats showed an enhanced response to heroin with an increased heroin intake tolerance when they grew up, suggesting that adolescent cannabis exposure increases the possibility of the use of heroin [59].

While more evidence suggests the gateway-based mechanism by which cannabis use leads to subsequent drug use, there are also studies suggesting a common liability model. The common liabilities may include genetics, personal sensitivities, and family factors [60]. In addition to physiological mechanisms, social and cultural factors also play important roles in the smoking of tobacco and cannabis [61]. By further comparing the influence of the environment and genetic factors with later cannabis use on a twin sample of 3,744 individuals, researchers revealed genetic factors to have the same impact on cannabis or other drug use [62]. More studies are needed to establish a link between cannabis and other drug use.

### 4.4 Effect of Cannabis Use on Education Achievement

A survey from New Zealand showed a clear association between increased levels of cannabis use at the ages of 14−21 and lower levels of academic degrees earned in adulthood, with implications for work, income, relationships, and more in future adult life [63]. Cannabis use during adolescence may also lead to a subsequent decline in academic performance in school, elevating the risks of dropouts [64]. These results have been linked to the induction of motivational syndrome or cognitive impairment [65]. Adolescents who were passively exposed to cannabis or have experienced second-hand exposure during pregnancy or infancy and continued to develop cannabis use have displayed lower average intelligence scores. Moreover, visual processing speed testing, a crucial form of measurement in the intelligence quotient (IQ) test [66], also displayed poor performances of cannabis users compared to non-users [67, 68]. However, when regular users stopped using it for 3 months, the adverse effects were no longer evident [67]. A study on 1,037 individuals from birth to adulthood between the ages of 13 and 38 revealed that those who used cannabis at age 13 and those who were still users at age 38 showed a neuropsychological decline, as well as a decline in cognitive and intelligence quotient. Even if the teenager later stops using cannabis, the damage remains irreversible [69]. The decline in IQ and learning ability directly affects the educational attainment and academic level of high school students [69, 70]. Further research demonstrates that teenagers who use cannabis, regularly or not, are the most meditative with their IQ declines. In contrast, the IQ of adult cannabis users does not exhibit significant change, regardless of the frequency of use [69]. This further illustrates the critical impact of cannabis on the development of the nervous system of adolescents, which in turn affects teenagers’ educational attainment and academic performances in school [69]. Furthermore, a 10-year study on 1,943 secondary school students in Australia showed adolescent cannabis use as associated with lower educational attainment, and therefore, cannabis use can affect students’ IQ and learning abilities, resulting in a decline in educational achievement and poor performance, which can also lead to more cannabis use in those teenagers [71, 72]. A longitudinal study on 1,265 children in New Zealand over a 25-year period highlighted the impact of the frequency of cannabis use, showing that the higher the frequency, the greater the chances of dropping out of school in teenagers. For instance, 16-year-olds exposed to cannabis are more likely to drop out than 20-year-olds, indicating that adolescents who are younger are more likely to drop out of school than adults. In addition, young people who regularly use cannabis are significantly less likely to enter college or earn a bachelor’s degree [73].

Although there are different views on the relationship between cannabis use and educational attainment, there are the following consensuses: 1) cannabis use affects intelligence and learning ability that leads to a decline in educational attainment; 2) low levels of educational attainment are likely to lead to an increase in cannabis use among adolescents; 3) there is no direct relationship between cannabis use and education, but it is determined by a combination of complex factors. To better observe this relationship, a study on 3,337 twins showed results of how male twins reported a higher rate of cannabis use than female twins, but cannabis use did not directly cause early school dropouts. However, genetic factors, family, and social factors combine to result in early cannabis use and lower educational attainment [74]. Limitations, such as the sample size, may prevent the study from completely disproving previous direct relationships between cannabis use and academic attainment; therefore, more in-depth research should be conducted in the future [74].

## 5 LEGAL STATUS OF CANNABIS IN THE UNITED STATES

**Fig. (3):**
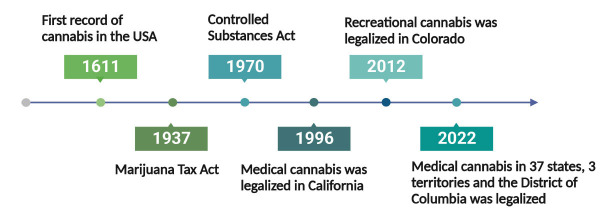
Historical policies of cannabis legalization. The legalization of cannabis has gone through many milestones since its introduction in the United States in 1611. It took decades from restricting the use of cannabis to the first legalization of medical cannabis in California in 1996, and then medical cannabis use was quickly legalized in 33 states across the United States.

When it comes to the legalization of cannabis, we must mention the emergence of cannabis-related laws and policies, such as marijuana laws in the United States [66]. Though the first record of cannabis in the United States dates back to 1611, cannabis was not legal for a long time despite many doctors prescribing cannabis for medical uses [75]. However, the medical or recreational use of cannabis was not explicitly restricted until the Marihuana Tax Act of 1937 [76]. Subsequently, the Controlled Substances Act of 1970 listed cannabis on Schedule I of a total of 5 classes of controlled substances. Restrictions were also placed on using cannabis medically and the cannabinoid derivatives, including all parts of the cannabis plant and its processed components [77]. Doctors could not prescribe medical cannabis to patients unless federally approved. It was not until 1996 that medical uses of cannabis were legalized for the first time in California [78], and more states followed the lead. Recreational cannabis, however, first became legal in 2012 in Colorado, but its sales were not legalized until 2014 [79]. As of 2022, the medical use of cannabis has been legalized in 37 states, 3 territories, and the District of Columbia (Fig. **3**) [80, 81]. Another 10 states only allow “low THC, high cannabidiol” products for special medical or legal defense measures [81].

As of today, many states have adopted corresponding laws to restrict or regulate cannabis, but nuances of these policies vary, nor are there uniform standards for the use of medical cannabis [82]. Legalizations of medical cannabis are primarily based on its therapeutic effects on pain, especially to help reduce the use of medical opioid analgesics [83, 84]. An analysis of a dataset of more than 1.5 million individual opioid prescriptions between 2011 and 2018 revealed that medical or recreational cannabis use reduced prescribed opioid use in terms of reduced frequency of use, number of patients, and probability [83]. Another study analyzing prescription drugs using Medicare Part D from 2012 to 2015 showed that patients took fewer daily opioids, such as hydrocodone and morphine, in states where medical cannabis was legal [85]. With respect to young people, medical cannabis is mostly used for patients above the age of 18 with severe or chronic pain, but it can also be used for minors under the age of 18 with consent from a guardian [86].

A previous study in 2014 has shown that the legalization of cannabis plays an important role in adolescent substance use [87]. As law enforcement approaches for cannabis control decline, our efforts to reduce negative outcomes of cannabis use in adolescents should increase. Recently, it has been demonstrated that the states with legal cannabis use have more adolescent cannabis users. A recent study in Oregon has found the rates of cannabis use in middle schools to be increased after the legalization of recreational cannabis, and the increased cannabis use in adolescents has been found to have a negative impact on students' academic achievement and mental health [88]. Additionally, the proportion of young adult criminal justice referrals to cannabis use disorder treatment has declined after the legalization of recreational cannabis use [89], which could indicate a potential health issue. Other referral sources may be included to enhance the treatment of cannabis use disorder in adolescents and young adults.

## CONCLUSION

To date, most studies have demonstrated that cannabis use causes detrimental harm to adolescents (Table **1**), though debate continues over this issue. While medical uses of cannabis may offer positive effects that could aid treatments for some diseases, adolescents should generally avoid using cannabis in a recreational manner. As more states in the United States legalize both medical and recreational use of cannabis, recreational cannabis is mostly still only available to people 18 years of age or older, which is cohesive to findings that suggest the main influences of cannabis on adolescent development. Results from current studies serve as guidance to individual decisions regarding cannabis use, and more preclinical and clinical studies are needed to provide detailed information for evidence-based decisions in the future.

## Figures and Tables

**Fig. (1):**
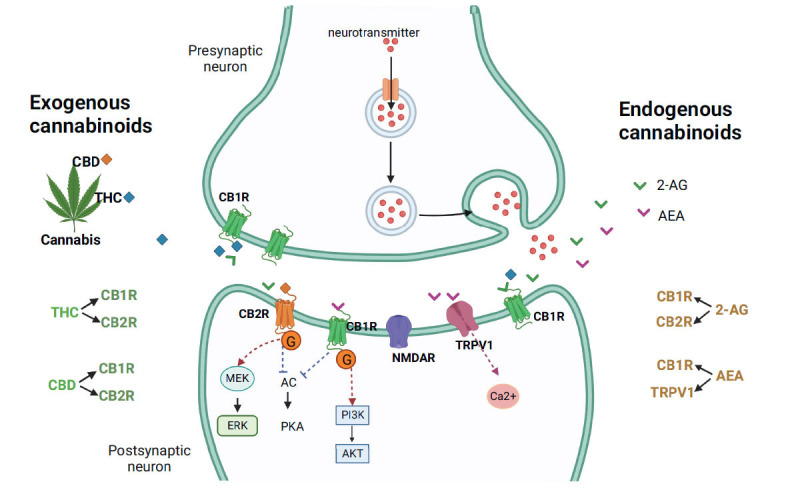
Exogenous and endogenous cannabinoid signaling. The endogenous cannabinoids include AEA and 2-AG, while endogenous cannabinoids mainly contain active ingredients of CBD and THC. CB1 and CB2 receptors can be activated by both exogenous and endogenous cannabinoids, which then inhibit AC and the downstream PKA signaling pathway by coupling with G protein. In addition, CB2 receptors mainly initiate MEK/ERK signaling after activation, while CB1 receptors mainly exert their effects through the PI3K/AKT signaling pathway. For endogenous cannabinoids, in addition to binding to CB receptors, AEA can also activate TRPV1 receptors. AC: adenylyl cyclase; AEA: anandamide; 2-AG: 2-arachidonoylglycerol; AKT: protein kinase B; CBD: cannabidiol; ERK: extracellular signal-regulated kinase; MEK: mitogen-activated protein kinase kinase; PI3K: phosphatidylinositol 3-kinase; PKA: protein kinase A; THC: tetrahydrocannabinol; TRPV1: transient receptor potential vanilloid.

**Fig. (2):**
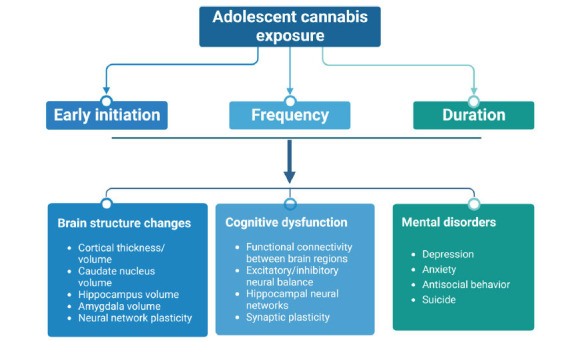
Effects of cannabis use on adolescent development. The onset, frequency, and duration of early exposure to cannabis in adolescents can affect brain development, alter cognitive function, and cause mental disorders.

**Table 1:** Studies on the neuropsychological outcomes caused by cannabis use in adolescents.

**Studies**	**Sample Size**	**Frequency of Cannabis Use**	**Age Range**	**Neuropsychological Outcomes and Other Appropriate Variables**
A 10-year prospective-longitudinal community study	1395	NA	14-17	Mood disorders, panic-anxiety and dysthymia; social phobia [24]
A prospective study in young people	34 653	High	18 and over	Behavioral disinhibition; significant increase in the prevalence and incidence of other drug use disorders, especially alcohol and nicotine [25]
Diffusion tensor imaging study	23	Heavy	18-20 (onset use before 17)	Brain structures change including superior longitudinal tact, parietal lobe, superior frontal gyrus and cingulate gyrus; poor performances in language learning [27]
Longitudinal study with fMRI scans	65	High amount use	10-21	Brain functional connectivity and cognitive development decrease; lower intelligence quotient [28]
Cohort study with fMRI scans	799	Dose-dependent use	14-19	Cortical thickness, neurodevelopment, and CB1 receptor decrease; attentional impulsiveness; other substance use (alcohol and nicotine) [29]
Cohort study with fMRI scans	82	NA	15-37	Brain volume reduction in Schizophrenia patients with cannabis use compared with patients without cannabis use; less improvement in psychotic symptoms [31]
Cohort study with fMRI scans	9498	Frequent use (≥3 times per week)	8-22	Left prefrontal cortex thickness reduction; lower estimated intelligence quotient [34]
Retrospective study about the onset of cannabis use age	68	Regular use (1-7 times/week)	12-35	Psychotic symptoms: depression, anxiety, depersonalization; distractibility; weakness of focused thinking [35]
A prospective study in young people	4568	Frequent use (≥3 times per week)	14-21	Mild executive and cognitive dysfunctions; higher levels of externalizing/behavior symptoms and lower levels of fear/phobia [38]
Experimental study with fMRI scan	111	Regular use (at least twice/month)	19-55	Potential damage to hippocampal structural and neurochemical integrity induced by THC instead of CBD [39]
Experimental study	2071	Frequent	6^th^ graders-20 years old	Consistently more emotionally impaired than occasional users and non-users [42]
Longitudinal study of child development	1606	Use weekly	15-20	Depression and cannabis use mutually enhancing; increased risk of suicidal ideation [43]
Birth cohort study	3239	Frequent (at least every few days)	14 and 21	Increased risk of AD in early childhood; anxious and depressive behavior; aggression and delinquency behavior; independent with other illicit drugs [45]
A longitudinal cohort study	2311	NA	11-24	Mild depressive behavior in males instead of females [47]
Birth cohort study	1003	Frequency dependent	0-25	Increased levels of cannabis use caused lower levels of academic degrees [63]
Cohort study	1037	Cannabis dependence at one, two, three or more waves	13-38	Impairment of global domains of neuropsychological functions; cognitive and intelligence quotient decline [69]
A 10-year longitudinal cohort study	1943	Occasional and weekly+ cannabis users	13-25	Depression/anxiety symptoms; alcohol use and cigarette smoking; lower educational attainment [71]
A 25-year longitudinal study	1265	More than 100 occasions by age 16	12-25	Indirect potential cognitive deficit due to social context environments; leaving school without qualifications; tendency of reduced success of obtaining university degrees [73]

**Note: ** NA, not applicable.

## LIST OF ABBREVIATIONS

2-AG: 2-arachidonoylglycerol
AEA: N-arachidonoyl Ethanolamine
CB1: Cannabinoid Receptor 1
CB2: Cannabinoid Receptor 2
CBD: Cannabidiol
DTI: Diffusion Tensor Imaging
fMRI: Functional Magnetic Resonance Imaging
IQ: Intelligence Quotient
THC: Tetrahydrocannabinol
TRPV1: Transient Receptor Potential Vanilloid 1
